# Long-Term Outcomes of Thermal Ablation for Benign Thyroid Nodules: The Issue of Regrowth

**DOI:** 10.1155/2021/9922509

**Published:** 2021-07-21

**Authors:** Jung Suk Sim, Jung Hwan Baek

**Affiliations:** ^1^Department of Radiology, Withsim Clinic, Seongnam 13590, Republic of Korea; ^2^Department of Radiology and Research Institute of Radiology, University of Ulsan College of Medicine, Asan Medical Center, Seoul 05505, Republic of Korea

## Abstract

Thermal ablation (TA) for benign thyroid nodules (BTNs) is widely accepted as an effective and safe alternative to surgery. However, studies on the long-term outcomes of TA have reported problems with nodule regrowth and symptom recurrence, which have raised the need for adequate control of regrowth. Therefore, a more complete TA with a longer-lasting treatment effect may be required. In this study, we review and discuss long-term outcomes and regrowth of BTNs following TA and evaluate factors affecting the long-term outcomes. We also discuss the management of regrowth based on long-term outcomes.

## 1. Introduction

Thermal ablation (TA) for benign thyroid nodules (BTNs) was introduced in the early 2000s with the clinical application of laser ablation (LA) and radiofrequency ablation (RFA), after which the efficacy and safety of TA were established [1, 2]. Since the 2010s, studies reporting >3 years of follow-up results of LA and RFA have been published [3, 4], and other modalities such as microwave ablation (MWA), bipolar RFA, and high-intensity focused ultrasound (HIFU) have begun to be used for the treatment of BTNs [5–9]. TA has now been widely used not only for the treatment of BTNs, but also for goiter, bilateral tumors, recurrent thyroid cancer, and papillary thyroid microcarcinoma [10–13].

The initially published studies reported relatively short-term follow-up, mainly of around 1 year, and usually considered successful treatment to be that achieving 50% or more volume reduction and symptom resolution [10]. However, with the publication of follow-up results over >2 years, nodule regrowth and symptom recurrence have been reported [14, 15]. Some patients showing recurrence were treated with additional TA, but some underwent surgery, which raises the need for adequate control of regrowth [16, 17]. Therefore, a more complete TA with a longer-lasting treatment effect is required as an alternative to surgery [18].

In this study, we review and discuss long-term outcomes and regrowth of BTNs following TA and evaluate the factors affecting the long-term outcomes. We also discuss the management of regrowth based on long-term outcomes.

## 2. Consensus of the Term “Long Term”

There is currently no consensus on the definition of “long-term follow-up”. Given that regrowth is clinically important after TA, the timing of regrowth can be a determinant of long-term follow-up [19]. According to previous studies, “long-term follow-up” varies from 2 to 5 years [3, 17, 20–23]. However, a meta-analysis and systematic review on the long-term outcomes of TA included studies with follow-up of >3 years [24, 25]. Regrowth is rarely mentioned in papers reporting results up to 1 year but is often mentioned in papers with follow-up periods of >2 years. Several studies reported a regrowth rate of 20% to 30% after RFA and LA, and regrowth appears to occur after 2 or 3 years, depending on the degree of complete treatment of the nodule margin [16, 17, 21, 26–28]. In this context, we suggest that 3 years is appropriate for the description “long term” in respect to the follow-up of BTNs treated with TA.

## 3. Definition of Regrowth

Various definitions of regrowth have been suggested, including a nodule volume increase of >50% compared with the minimum recorded volume [29, 30], a nodule volume 20% larger than the volume at 1 year after treatment [21], and volume increase over the initial nodule volume [26]. The definition achieving the biggest consensus to date is a nodule volume increase of >50% compared with the minimum recorded volume. We strongly suggest that a definition with full agreement is necessary for the following reasons: (1) standardization of regrowth reporting, (2) validation of regrowth rates, and (3) development of a management plan for patients with regrowth.

Recurrence has been used interchangeably with regrowth [23, 31]; sometimes, it is used to mean that the nodule volume has increased again [4, 14, 32], whereas other times, it is used to mean that the symptom has reappeared [17].

**Figure 1:**
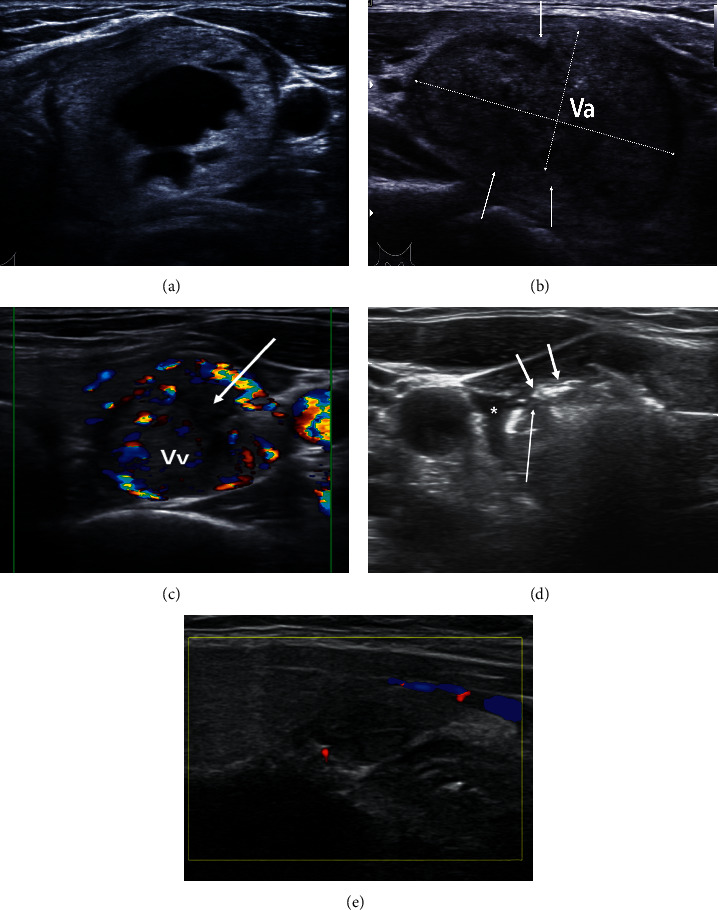
A benign symptomatic thyroid nodule in a 36-year-old female treated with three sessions of radiofrequency ablation (RFA). (a) The nodule was in the patient's left lower lobe. The longest diameter was 4.7 cm, and the volume was 20.6 mL. There was no vascularity in and around the nodule. (b) Longitudinal image of the RFA-treated nodule 1 month after the procedure. The total nodule volume (*Vt*) was reduced to 14.5 mL and the longest diameter to 3.8 cm. The volume reduction ratio was 30%. Ablated tissue located in the central portion of the treated nodule (*Va*) is surrounded by the peripherally located small amount of remaining viable tissue (arrows). The dotted lines indicate the measurements for *Va*, which best represent the volume of the complex and irregularly shaped ablated area. There was no vascularity. At this time, *Va* was 11.2 mL, the viable volume (*Vv*) was 3.2 mL, and the initial ablation ratio was 84%. (c) Color Doppler image of the treated nodule 19 months after the procedure shows the development of vascularity in the nodule. *Vt* had decreased to 6.8 mL, but *Va* (arrow) had regressed to 1.1 mL and *Vv* had increased to 5.7 mL. Such a *Vv* increase is an early sign that can predict regrowth, even while *Vt* is decreasing. As regrowth was expected, the patient received a second RFA in the following month. (d) Thirty-four months after the second RFA, the nodule showed a *Vv* increase again, and a third RFA was performed. Five percent dextrose injected for hydrodissection can be seen as an anechoic area between the nodule margin and carotid sheath (^*∗*^). The tip of the electrode (long arrow) is located near the veins of the nodule margin to achieve venous ablation. Air bubbles formed by ablation are compactly filling the venous lumen (arrows). (e) Ten months after the third RFA, the nodule had turned into a small scar-like region of tissue without vascularity. The *Vt* was 0.4 mL with no demonstrable *Vv*.

An early sign of regrowth was proposed by Sim et al. [16]. Nodules treated with TA were typically composed of two areas: a centrally located hypoechoic ablated volume (*Va*) and a peripherally located viable area, which is an undertreated area surrounding the *Va*. The total nodule volume (*Vt*) is the sum of the *Va* and viable area volume (*Vv*). *Vv*, which is practically impossible to measure, can be calculated by the formula *Vv*=*Vt* − *Va* (Figure 1). Regrowth is typically the result of the growth of the *Vv*. However, as *Vv* is generally small, it has little influence on change in *Vt* shortly after the procedure, whereas *Va* has a great influence on change in *Vt* as it is generally absorbed quickly in the year following TA. Therefore, even if *Vv* regrowth occurs within 2 years after the procedure, it can be offset by *Va* absorption. Thus, if *Vv* is traced separately, then regrowth may be detected earlier, even when *Vt* is decreasing (Figure 1). A *Vv* increase may be an early sign of regrowth and can be considered a predicting factor of regrowth; it was reported that a *Vv* increase precedes regrowth by 1 year [16].

It is worthwhile emphasizing that repeat cytology or biopsy is recommended because nodule regrowth can be a potential sign of overlooked malignancy [4, 19]; however, its value is debatable. Ha et al. [33] revealed that BTNs showing regrowth after RFA did not show cytomorphological alteration or any malignant transformation on biopsy.

## 4. Factors Related to the Long-Term Outcomes: Nodule Factors

Regrowth from marginal undertreated tissue, which is usually observed 2 or 3 years after the procedure, is closely related to long-term outcomes [34]. The baseline nodule volume influences both the regrowth rate and long-term outcomes [35]. TA can be used for symptomatic and cosmetic improvement of diffuse and/or multinodular goiter, but these uses have a different context from this review [12, 13]. Vascular nodules are resistant to TA because they disperse the input energy [36]. During follow-up, the development of vascularity can lead to regrowth [37]. Nodules with a cystic component and spongiform nodules have a tendency to show a greater volume reduction ratio (VRR) over both the short term [36, 38] and long term [32].

### 4.1. Baseline Nodule Volume

The baseline nodule volume is a major variable affecting long-term outcomes in respect to achieving a VRR >50% after 1 year, the regrowth rate, and normalization of thyroid function [17, 23]. The results of several studies support this view, with the larger the baseline nodule volume, the higher the regrowth rate and the lower the VRR. Some researchers have argued that there is no correlation between baseline nodule volume and outcomes, but studies making this claim appear to be low in number and present an inferior quality of evidence [21, 26, 32, 39].

Studies finding that the baseline nodule volume is associated with VRR are more numerous. Lim et al. [4] reported that, for nodules with a baseline volume < 10 mL, the final VRR was 94.5% after an average of 1.7 treatments, whereas nodules larger than 20 mL had a final VRR of 88.2% after an average of 3.8 treatments. Their multiple linear regression analysis showed that initial nodule volume (*P* < 0.001) was an independent factor predicting the final VRR. However, appropriate volume reduction can be achieved in larger thyroid nodules through the use of more treatment sessions. Deandrea et al. [23] showed that nodules with a volume < 10 mL were reduced by 82%, whereas nodules with a volume between 10 and 20 mL and those with a volume > 20 mL were reduced by 75% and 65%, respectively.

Bernardi et al. [17] found associations between baseline volume and retreatment; for RFA, a baseline volume of 22.1 mL and a 1 year VRR < 66% predicted retreatment, whereas for LA, a baseline volume of 14.5 mL and a 1 year VRR < 54% predicted retreatment. However, they did not find a clear association between baseline volume and regrowth.

Gambelunghe et al. [35] and Cesareo et al. [40] reported that TA was effective for treating autonomously functioning thyroid nodules, especially when the baseline volume was small and that nodule volume seems to be a significant predictive factor of the efficacy of TA. A meta-analysis also indicated that the baseline nodule volume was associated with the rate of thyroid-stimulating hormone normalization [41].

### 4.2. Vascularity

Nodule vascularity influences the VRR, regrowth rate, and long-term outcomes [9, 15, 36]. Blood vessels in nodules disperse the heat generated by ablation devices, creating the so-called heat-sink effect [2]. Ahn et al. described the vascularity of marginal viable tissue as a factor influencing volume reduction [42]. Moreover, vascularity is also a factor influencing regrowth [37]. During follow-up, if a nodule shows or develops vascularity, it has considerable potential for regrowth [14, 43]. Yan et al. reported that vascularity was an independent factor associated with regrowth [36], and Wang et al. reported that a patient group showing regrowth demonstrated more vascularity than a nonregrowth group [44].

## 5. Factors Related to Long-Term Outcomes: Technical Factors

Long-term outcomes differ depending on the modality used [24] and the energy delivered [17]. As it is not possible to ablate all nodule tissues in a single session of TA, a better long-term outcome can be achieved by applying a multiple-session treatment strategy [18]. Recently, techniques targeting the margin control have been introduced [9].

### 5.1. TA Modalities

Most studies comparing modalities report that VRR is influenced by the TA modality [45–47]. Although LA and RFA are generally known to be very effective and safe, prospective studies comparing them are currently limited. A randomized open-label parallel trial comparing LA with RFA at 6 months was reported. In this study, Cesareo et al. [45] concluded that RFA achieved a larger nodule volume reduction than LA. However, Mauri et al. [48] reported that RFA and LA are similarly feasible, safe, and effective for treating BTNs. RFA is faster than LA but requires significantly higher energy. In a Bayesian network meta-analysis, RFA achieved better VRR than LA (77.8% vs. 49.5%) [47]. Two meta-analyses and systematic reviews that compared the long-term outcomes of RFA and LA reported that the final VRR of RFA was higher, at 92.2% vs. 43.3% at 3 years or more and 87% vs. 44% at 3 years [24, 25]. The Italian minimally invasive treatments of the thyroid group reported that both RFA and LA resulted in significantly reduced nodule volume but that RFA was superior to LA [17].

Ha et al. [47] and Cho et al. [24] claimed that the difference between RFA and LA in the final VRR depends on whether marginal undertreated tissue is controlled. RFA uses a moving-shot technique with internally cooled electrodes. Because of its high maneuverability, this device can ablate marginally located nodule tissue as much as possible while minimizing thermal damage to surrounding tissues [2, 49]. By contrast, with the laser delivery fibers used in LA, ablation of the margin may be incomplete because the range of treatment is concentrated in the center of the nodule [26].

There are reports of LA outperforming RFA at 1 year follow-up. In a propensity score matching analysis, Pacella et al. [50] showed mean nodule reduction at 12 months of 70% ± 19% in an LA group vs. 62% ± 22% in an RFA group. They mentioned that the operator's skills could be crucial in determining the extent of nodule volume reduction, regardless of the technique used. Ben Hamou et al. [51] reported that the nodule volume had significantly decreased by 75.0% in an RFA group and 83.9% in an LA group at 18 months.

MWA and HIFU have only recently begun to be used, and long-term follow-up results are, therefore, rare. Monpeyssen et al. reported a VRR after HIFU of 33.3% at 2 years and 31.9% at 3 years [52], while Lang et al. [31] and Trimboli et al. [53] reported an average VRR at 2 year follow-up of 70.4% and 43.3%, respectively. HIFU has the advantage of being a truly noninvasive technique that does not require the insertion of electrodes or fibers through the patient's skin [52] but has the disadvantage of requiring a deeper anesthesia or sedation level because it is more painful than RFA or LA [54]. To our knowledge, there has been no report of the results of MWA or BTNs after >2 years of follow-up. Articles comparing the efficacy of RFA and MWA after a 1 year follow-up period reported that the results were similar [46, 55].

### 5.2. Energy Delivered

Another factor affecting long-term outcomes is the energy delivered. Bernardi et al. [17] reported that RFA with a cutoff of 1360 J/mL predicted VRR > 50% and RFA with a cutoff of 918 J/mL predicted retreatment. Trimboli et al. [56] found that the energy delivered was significantly correlated with VRR in nodules < 10 mL at 1 year, and Deandrea et al. [57] demonstrated that delivering 756 J/mL and 2670 J/mL gave a probability of VRR > 50% in 50% and 99% of patients, respectively, at 1 year.

### 5.3. Multiple-Session Ablation

Additional VRR is gained through multiple-session treatment [4]. However, sufficient effects can be obtained only when the method and timing of the additional treatment are selected appropriately [16, 47]. Huh et al. reported a randomized trial comparing single-session vs two-session RFA for benign nodules. Their results showed that single-session RFA achieved significant volume reduction and a satisfactory clinical response in most patients, but that additional RFA was effective in patients with a large nodule (>20 mL) or unresolved clinical problems [58]. Deandrea et al. reported no VRR gain from one or two lots of additional LA [3]. In a systematic review and meta-analysis of the efficacy of TA for benign nonfunctioning solid thyroid nodules, Trimboli et al. reported no significant difference in VRR between single- and multiple-session LA [25].

However, contrasting results have also been reported. Lim et al. achieved >90% VRR after 4 years through multiple-session RFA [4], and in their meta-analyses and systematic reviews, Ha et al. and Cho et al. found that the long-term VRRs of RFA were higher than those of LA. The authors argued that the reason the final VRR of RFA was higher is that it could effectively control marginally located viable tissue with multiple-session treatment [24, 47].

As in the report of Huh et al., when a nodule is large or clinical problems are not resolved, additional ablation gains further efficacy [58]. From our point of view, the differences in reported results are based on whether marginal undertreated tissue could be effectively controlled or not, and the main reason for differences in efficacy is the maneuverability of the device.

### 5.4. Recently Introduced Techniques for Margin Control

For it to be a comparable alternative to surgery, TA has to achieve sustainable efficacy, such as life-long problem resolution. For this, some advanced techniques were recently introduced [59]. Park et al. proposed an advanced RFA technique to control marginal regrowth that they named vascular ablation. Two different vascular ablation techniques are available: artery-first ablation and marginal venous ablation. The artery-first ablation technique can be applied to hypervascular thyroid nodules with a prominent feeding artery. However, the marginal venous ablation technique is useful for most thyroid nodules because thyroid nodules usually have marginal draining veins [9]. Offi et al. reported that the VRR of RFA after ablation of the feeding artery was higher than that of RFA using the conventional technique [36].

Hydrodissection is another important technique for improving safety during margin ablation. Injecting 5% dextrose between the nodule margin and the surrounding tissues can create a space that helps in achieving complete ablation of marginal nodule tissue (Figure 1) [9].

## 6. Conclusions

TA for BTNs is effective and safe according to the reports of short-term follow-up studies, but as long-term outcomes have been announced, it is becoming evident that the problem of regrowth is important. We should understand that regrowth from marginal undertreated tissue is frequently reported 2–3 years after TA. To minimize regrowth, complete ablation of the nodule margin using advanced techniques and an understanding of the influencing factors are necessary. In patients with large nodules or incompletely resolved clinical symptoms, additional treatment is required to achieve a long-term satisfactory effect. We hope that this review may help establish TA as a long-lasting treatment method with comparable outcomes to surgery in clinical practice.

## Data Availability

The data used to support the findings of this study are included within the article.
